# The Unknown Risk of Vertical Transmission in Sleeping Sickness—A Literature Review

**DOI:** 10.1371/journal.pntd.0000783

**Published:** 2010-12-21

**Authors:** Andreas K. Lindner, Gerardo Priotto

**Affiliations:** 1 London School of Hygiene & Tropical Medicine, London, United Kingdom; 2 Epicentre, Paris, France; Institute of Tropical Medicine, Belgium

## Abstract

**Background:**

Children with human African trypanosomiasis (HAT) present with a range of generally non-specific symptoms. Late diagnosis is frequent with often tragic outcomes. Trypanosomes can infect the foetus by crossing the placenta. Unequivocal cases of congenital infection that have been reported include newborn babies of infected mothers who were diagnosed with HAT in the first 5 days of life and children of infected mothers who had never entered an endemic country themselves.

**Methods:**

This review systematically summarizes the literature on the vertical transmission of HAT, to our knowledge for the first time. To approach the broader aspects of the subject, articles considering the epidemiology of childhood HAT and HAT in pregnancy were also included. The HAT guidelines and technical reports of the World Health Organisation, Médecins Sans Frontières, Institut de Recherche pour le Développement, and of one endemic country were reviewed.

**Results:**

Publications describing congenital HAT are very limited and consist only of single case reports and small case series. Generally it is assumed to be a rare event, but it has never been systematically investigated. In two publications, it is hypothesized that congenital HAT occurs more often than suspected. Not all guidelines and not all HAT literature mention this transmission route.

**Conclusions:**

The risk of vertical transmission is unknown. Awareness of congenital HAT is insufficient, and as a result opportunities for an early diagnosis in newborns may be missed. All HAT guidelines and local HAT protocols should stress that in endemic areas pregnant women should be systematically checked for HAT and that newborns of HAT infected mothers should be assessed for the disease as soon as possible. Studies on the impact of HAT on fertility and pregnancy and studies on congenital HAT are long overdue.

## Introduction

Human African trypanosomiasis (HAT), commonly known as sleeping sickness, is considered as invariably fatal if left untreated. The infection with the protozoan parasite *Trypanosoma brucei gambiense* (in western and central Africa) progresses over a few months to several years from the haemolymphatic first stage to the meningoencephalitic second stage. *Trypanosoma brucei rhodesiense* (in eastern and southern Africa) generally causes a more acute form. Currently, 97% of all reported cases are caused by *T.b. gambiense* [1].

By 1960, HAT had been reduced to a very low level after large-scale control efforts sustained during many decades. However, shortly after these efforts were reduced or even abandoned in the early 1960s, HAT reemerged in several countries, reaching a peak in the mid-1990s. Since that time, the efforts of the World Health Organization (WHO), national control programmes, bilateral development cooperation, and non-governmental organisations have significantly reduced the burden of sleeping sickness [1]. Nevertheless, it has become increasingly difficult to obtain sufficient funding to sustain adequate control efforts. In 2006, although about 12,000 cases of HAT were reported by disease-endemic countries, WHO estimated the cumulative number of people infected with HAT at 50,000 to 70,000 [2]. The disease strikes in remote areas and affects marginalized and poor communities. Its spread is amplified in areas of chronic conflict, where weak health systems and political instability interfere with prevention and control.

HAT belongs to the group of neglected tropical diseases. Neglect can also be seen in the scarcity of research and development for new diagnostic tools or therapeutic agents. Although extensive basic scientific research has been done about the trypanosomes, clinical research has been neglected [3]. Concerning HAT, Stich et al. state that “the investment in clinical and applied aspects of research into the disease is so dire that even small amounts of additional funding are likely to produce disproportionately large returns” [3].

Children with HAT present with a range of generally non-specific symptoms [4]–[7]. Misdiagnosis and late diagnosis of HAT is frequent with often tragic outcomes, such as brain damage resulting in physical and mental sequelae or death. In pregnant women, trypanosomes can infect the foetus (vertical transmission), which is generally considered to be a rare event [8]–[12]. In the following, the literature concerning this transmission route is reviewed in regard to the following questions: How often is congenital HAT described in the literature? What is the risk of congenital HAT? What is the epidemiological impact of congenital HAT?

## Search Strategy and Selection Criteria

The electronic database PubMed was searched with the keywords “African trypanosomiasis”, “sleeping sickness”, or “T. brucei” in all possible combinations with “vertical transmission”, “congenital”, “neonatal”, “newborn”, “infant”, “child*”, or “pregnan*” (asterisk “*” as truncation symbol). The search was neither limited by study design nor by language of publication, nor by date of publication.

To approach the broader aspects and implications of vertical transmission, articles considering the epidemiology of childhood HAT and HAT in pregnancy were also searched for.

WHO Web sites were searched for relevant publications. A technical report from WHO [13], guidelines from Médecins Sans Frontières (MSF) [8], Institut de Recherche pour le Développement (IRD) [14], and one endemic country [15], and the latest editions of two standard textbooks in tropical medicine [10], [16], were reviewed.

Initially, titles and abstracts were screened. Articles identified as possibly relevant were reviewed as full text. The reference lists of included articles were assessed for further relevant publications. All publications describing congenital HAT were included in this review.

## Results and Discussion

### Diagnosis of Congenital HAT

Most authors define congenital infection as the diagnosis of HAT in a newborn of an infected mother within the first 5 days of life [5], [11], [12], [17], [18]. This definition assumes that after a bite by a tsetse fly, the dissemination of trypanosomes from the inoculation site into the bloodstream is expected to take more than 5 days. In the literature, different intervals are proposed before the presence of trypanosomes in the blood can be demonstrated, for example 5 to 21 days [9] or 7 to 10 days [11]. Chambon hypothesized that the period of dissemination into the bloodstream may be reduced in young infants [19].

Furthermore, certain vertical transmission can be assumed in children of an infected mother who are diagnosed with HAT and never entered an endemic country themselves [7], [11].

The diagnosis of HAT in newborns should be established through direct parasite detection. Serological screening with the card agglutination test (CATT) is considered less reliable in newborns. The presence of maternal antibodies may lead to false positive results. An immature immune response in newborns may yield false negative results [8].

### Limited Publications Describing Congenital HAT

Publications describing congenital HAT consist only of single case reports or small case series. Since 1933, only 13 cases of congenital infection with *T.b. gambiense*—diagnosed within 5 days of life—are described in the literature [5], [17], [19]–[24]. One case of congenital infection with *T.b. rhodesiense* of a 2-day-old newborn is reported [25].

The largest case series was published by Triolo et al. in 1985 [5]. Six cases of congenital HAT were diagnosed within 5 days of birth by direct parasite detection in the hospital of Fontem, southwest Cameroon. All six were in the second stage of disease (three with trypanosomes in the cerebrospinal fluid). Of these six newborns, three were treated with Melarsoprol and left the hospital apparently cured. Three died soon after birth before a treatment could be started. With regard to the six mothers, five were in the first stage with normal cerebrospinal fluid. This suggests rapid infection and disease progression for the child in utero. Yet, the duration of pregnancy is sufficient to allow a normal progression to second stage disease in utero. The six cases of congenital HAT were reported among 227 children under 6 years old diagnosed with HAT during an observation period of 17 years. Therefore, congenital HAT comprised 2.6% of the cases in children under 6 years old.

The data on the first three of the above mentioned six cases were already published in 1979 by Sina et al. from the same study group. Sina et al. emphasized that these three cases were diagnosed in a relatively short period of some few months in a hyperendemic area. Along with these three infected newborns, two cases of uninfected newborns from infected mothers were reported [17].

Kalanda et al. described a twin birth from a mother suffering from first stage HAT [24]. In the first twin, parasites could be detected in the blood and second-stage congenital HAT was diagnosed. The second twin was followed up and 6 weeks after birth, the serological testing became positive and the cell count of the cerebrospinal fluid indicated second stage HAT. However, the second twin did not fulfill the criteria of congenital HAT. Both twins showed normal results at the 6 months post-therapeutic follow up.

David and Pape reported two newborns—tested immediately postpartum—with trypanosomes in the peripheral blood and in the umbilical cord blood. Their mothers appeared healthy and had been diagnosed through routine testing [20].

Chambon, Pinto, Burke et al., and Kazyumba et al. each published a single case of congenital HAT diagnosed in the first 5 days of life [19], [21]–[23].

Three cases of children (19, 22, and 24 months old) with second-stage HAT—born in France and England of infected mothers—are described. The three children themselves are reported not to have been in an endemic country, and vertical transmission can be assumed [26]–[28]. For example, one of these children was born with hydrocephalus and second-stage HAT in Marseille, France (the mother left Chad in the fifth month of her pregnancy [26]).

The proportion of uninfected babies born to infected mothers is unknown. Besides the two uninfected newborns described by Sina et al., Darré refers in his publication in 1937 to four cases of uninfected newborns from infected mothers in a study published by Thiroux, Lebœuf, and van den Branden [17], [26].

In summary, 17 cases of certain vertical transmission could be identified in the literature: 13 cases with *T.b. gambiense* and one case with *T.b. rhodesiense* diagnosed within 5 days of life, plus three children of infected mothers, who were diagnosed with HAT and never entered an endemic country themselves.

### Uncertainty of Transmission Route in Older Newborns

Chambon refers in his publication in 1933 to four cases of HAT in newborns diagnosed at the age of 8 to 12 days observed by Le Rougic, Montestruc, Montalier, and Jamot [19]. Several other publications reported children diagnosed beyond 5 days of life in the newborn period or as very young infants and the authors assumed vertical transmission [11], [18], [29]–[37]. For example, Pepin et al. stated that one or two cases of congenital transmission among 200 to 400 total cases per year were treated [18]. In this publication the authors refer to two infants 1 and 3 months old. However, postnatal infection cannot be excluded in these cases. The older the child is at the time of diagnosis, the higher the probability of vector transmission. With increasing age other risk factors, like shared exposure with the mother, become more plausible.

### Higher Proportion of Second-Stage HAT in Younger Children

In general, HAT is diagnosed less in younger children than in adults [4], [5]. In the aforementioned study of Triolo et al. in Cameroon, out of 227 children under 6 years with HAT, 46 (20.3%) were infants under 1 year old [5]. In a recent study by Eperon et al. in Sudan, of 119 infected children under 6 years, 42 (35.3%) were under 1 year old [4]. Assuming an increasing incidence of vector-transmitted disease with age (longer exposure to the vector), in those two studies children under 1 year seemed to be overrepresented.

Among the 119 children under 6 years in Sudan, the proportion of children in the second stage was higher in very young children (under 2 years) compared with older children [4]. Even if different second-stage criteria in regards to the white cell count in the cerebrospinal fluid complicate the comparison, the predominance of second-stage HAT in young children is reported in several other studies as well [5], [11], [38], [39]. Late diagnosis due to an unspecific initial clinical presentation is a possible explanation [4]–[7]. A more rapid progression from first to second stage is discussed by several authors [4], [5], [7]. In very young children, more than five white blood cells/mm^3^ may occur in the cerebrospinal fluid without any pathological process, leading to some over-classification as second stage [4], [40], [41].

The higher proportion of second-stage HAT in younger children points to an infection early in life, and vertical transmission may be a contributing factor.

### Familial Clustering of HAT

Familial aggregation of HAT had already been observed at the beginning of the 20th century [42]. Méda et al. showed familial clustering in a case control study in Ivory Coast [43]. Khonde et al. demonstrated, in three adjacent villages in Democratic Republic of Congo, familial clustering between mothers and children, and between brothers and sisters [44]. Khonde et al. discuss behavioural factors, with a shared exposure as the most plausible explanation for familial clustering of HAT. For example, breastfeeding mothers usually carry their infants with them and so both are simultaneously exposed to an infective tsetse fly. They do state that “congenital infection may have been involved in some cases”. Familial aggregation is also described in two case-control studies in *T.b. rhodesiense* [45], [46].

### The Unknown Prevalence of HAT in Pregnant Women

Women with HAT have increased infertility and abortion rates [47]. Among 160 women of childbearing age infected with *T.b. gambiense*, Sina et al. reported 53 (33%) women with hormonal disorders, especially amenorrhoea. Furthermore, among them, ten abortions and seven normal pregnancies were observed [17]. Among 76 women of childbearing age with *T.b. rhodesiense* HAT, Buyst et al. described four pregnant women with complications such as hydramnios, preeclampsia, abortion, stillbirth, and premature birth in an observation period of 13 months [25].

No published data about the prevalence of HAT in pregnant women in endemic areas could be found. HAT screening is not integrated into antenatal care services.

Very few data are available about the prevalence of pregnancy among treated HAT patients. In two databases in Yei and Kiri, South Sudan, pregnancies among infected women of childbearing age were 2.8% (17/606) and 0.7% (8/1191), respectively [48].

However, those retrospective data may considerably underestimate the reality. Pregnancy testing is rarely performed on a routine basis on HAT patients and early pregnancy may be missed. HAT can be asymptomatic or a little symptomatic in pregnant women [26], [37], and may therefore not be detected in antenatal care services. Infected pregnant women may await their delivery at home and present to the health facility for HAT diagnostics afterwards, resulting in underreporting of abortions, stillbirths, and early deaths in congenital HAT cases.

### Lack of Consistent Databases

HAT databases that were consulted did not routinely record the pregnancy status of infected women. Furthermore, the link between infected mother and the status of her newborn or infant could not be established.

If a newborn is diagnosed, the age is usually recorded in months. As a result, even a case diagnosed in the first 5 days of life is likely to be recorded with the age of 1 month. Due to these limitations, relevant information about congenital HAT cannot be drawn from existing databases of which we are aware.

### Lack of Awareness

As part of a master thesis at the University of Basel, Switzerland, 18 international and local HAT experts were interviewed [48]. Among other things, they were asked if, according to their experience, newborns of treated breastfeeding HAT patients were followed up. Five experts stated that newborns were followed up. Eight experts acknowledged that newborns were followed up if possible. However, four experts declared that newborns were not followed up at all.

Vertical transmission of HAT is not mentioned in several examples of HAT literature, such as a standard textbook of tropical medicine and a clinical HAT review [3], [16].

National HAT guidelines for endemic countries are scarce. The Médecins Sans Frontières guideline clearly points out this transmission route and recommends testing of newborns of infected mothers [8]. A WHO technical report mentions that newborns of infected mothers are at risk, but does not specify any recommendations for screening activities [13]. A reviewed national guideline for an endemic country did not mention the vertical transmission route [15]. The occurrence of familial clustering of cases with consequent recommendations for active or passive case-finding was not pointed out in the three aforementioned documents. The guideline of Institut de Recherche pour le Développement does not describe the vertical transmission route, although it discusses the occurrence of familial clustering [14].

### Recommendations for HAT Guidelines

All HAT guidelines and local HAT protocols should put emphasis on diagnosing HAT in pregnant women, newborns, and infants (Box 1).

**Box 1. Six Aspects Should Be Stressed in All HAT Guidelines and Protocols**

- Newborns of mothers with HAT should be assessed by parasitological methods as early as possible (including umbilical cord blood examination where possible).
- In case of initial negative parasitological results, newborns of mothers with HAT should be followed up for at least 6 months.
- In endemic areas pregnant women should be screened for HAT.
- Women with HAT of childbearing age should undergo pregnancy testing.
- Appropriate treatment protocols for infected newborns and infants should be established.
- Familial clustering of cases should be considered in all active and passive case-finding strategies.

The sensitivity and specificity of CATT for infants under 6 months is not known. If suspected of HAT infection, children in this age group should undergo direct parasite detection exams. Newborns of infected mothers initially yielding negative results should be followed up for at least 6 months. This is a reasonable approach due to the limited sensitivity of direct parasite detection techniques routinely used in the field [49].

In endemic areas, HAT should be considered in the differential diagnosis of newborns and infants. The existence of familial clustering should be borne in mind in all active and passive case-finding strategies. Especially in the case of an infected mother, the higher risk of infection of all her children has to be considered. Screening should be offered to the whole family. This is particularly relevant in passive case detection, where only an individual is tested. In active case-finding, relatives of cases who did not attend the community screening should be systematically sought [44].

### Vertical Transmission Underestimated?

Most case reports of congenital HAT are from the 1970s and 1980s. The resurgence of HAT from the 1960s onwards and its peak in the mid-1990s did not result in more reported cases, although more sensible screening and diagnostic methods had become available. Thirteen cases of congenital infection with *T.b. gambiense* diagnosed within 5 days of birth are described in the literature. It is less surprising than it would appear at first sight that nearly half of all cases were diagnosed at the hospital of Fontem in Cameroon [5], [17]. Triolo et al. pointed out that at Fontem there was a co-existence of favourable conditions: a well-functioning hospital and laboratory, the systematic performance of paediatric examination during Mother and Child Care clinics, and the symbiosis between the hospital and the school, which were both managed by the same mission congregation. Sina et al. drew attention to the fact that mass campaigns for HAT control leave little time for research. It should be borne in mind that in several countries—e.g., Democratic Republic of Congo—HAT screening and treatments were organized at the village level, far from hospitals, maternities, and well-functioning laboratories. The probability of detecting congenital HAT under those circumstances was extremely low. Sina et al. were testing the umbilical cord blood of newborns of mothers either with HAT or suspected HAT, or those who were resident in endemic areas. Using this method, they were able to detect three cases of congenital HAT in a period stated as several months. According to Triolo et al. and Sina et al., it appears that vertical transmission of HAT occurs more often than suspected.

In the limited publications on the subject, congenital HAT is assumed to be rare. Behavioural and spatial risk factors shared with the mother are a plausible reason for the frequency of infected infants. However, studies about congenital HAT are absent and the real risk is unknown.

### To Investigate the Unknown Risk

Is congenital HAT rare because infected women rarely give birth, due to infertility and abortion? Are babies born to infected mothers mostly exempt from infection? Or is congenital HAT occurring more often than suspected?

Routine data collection may be a relatively easy step to start obtaining relevant information. For diagnosed newborns, recording the age in days would better inform the data analyses. The outcome of pregnancy of infected women could be recorded as well as the HAT status of their newborns.

To assess the true association of vertical transmission with abortion, stillbirth, and congenital HAT, further research is needed. The mechanisms of transplacental passage of trypanosomes should be investigated as well. Studies on HAT complications during pregnancy and on congenital HAT are long overdue and should be promoted despite the often challenging context of remote areas, political instability, and poorly performing health services.

**Key Learning Points**

- Seventeen cases of certain vertical transmission could be identified in the literature, plus many more suspected, for whom vector transmission could not be excluded.
- Generally it is assumed to be a rare event, but it has never been systematically investigated.
- In two publications it is hypothesized that congenital HAT occurs more often than suspected.
- Awareness of this transmission route is insufficient and not enough effort is allocated to detect vertical transmission of HAT.
- In endemic areas, pregnant women should be screened for HAT. Newborns of mothers with HAT must be assessed with parasitological methods as early as possible and followed up for at least 6 months if initial results are negative.

**Five Key Papers in the Field**

- Triolo N, Trova P, Fusco C, Le Bras J (1985) [Report on l7 years of studies of human African trypanosomiasis caused by *T. gambiense* in children 0–6 years of age]. Med Trop (Mars) 45: 251–257.
- Sina G, Testa G, Triolo N, Trova P, Cramet B (1979) [Some new cases of congenital human African trypanosomiasis (*T. gambiense*) (author's transl)]. Med Trop (Mars) 39: 57–63.
- Chambon M (1933) Trypanosomiase humaine observée chez un enfant de mois de cinq jours. Bull Soc Path Exot 26: 607.
- Darré H, Mollaret P, Tanguy Y, Mercier P (1937) Hydrocéphalie par trypanosomiase congénitale. Démonstration de la possibilité du passage transplacentaire dans l'espèce humaine. Bull Soc Path Exo 30: 159.
- Debroise A, Debroise-Ballereau C, Satge P, Rey M (1968) [African trypanosomiasis in young children]. Arch Fr Pediatr 25: 703–720.

## Supporting Information

Alternative Language Abstract S1 Translation of the Abstract into French by Gerardo Priotto
(0.07 MB RTF)
