# Supplementary material for: The Unknown Risk of Vertical Transmission in Sleeping Sickness—A Literature Review
Source: PLoS Negl Trop Dis. 2010 Dec 21;4(12):e783. doi: 10.1371/journal.pntd.0000783 (PMC3006128; doi:10.1371/journal.pntd.0000783)
Supplement: Alternative Language Abstract S1 — Translation of the Abstract into French by Gerardo Priotto (0.07 MB RTF) [file pntd.0000783.s001.rtf]

Le Risque Inconnu de Transmission Verticale de la Maladie du Sommeil – Revue de la Littérature 
Andreas K. Lindner a * et Gerardo Priotto b

a London School of Hygiene & Tropical Medicine, Keppel Street, London WC1E 7HT, UK
b  Epicentre, 8 rue Saint-Sabin, 75011 Paris, France
* Correspondance,, E-Mail: A.K.Lindner@gmx.de

Résumé
Contexte: La trypanosomiase humaine africaine (THA) présente chez l'enfant une multitude de symptômes généralement non spécifiques. Un diagnostic tardif est fréquent, avec des conséquences souvent tragiques. Les trypanosomes peuvent infecter le fœtus en traversant la barrière placentaire. 
Des cas rapportés d'infection congénitale incontestable incluent des nouveau-nés de mères infectées avec un diagnostic de THA fait dans les premiers cinq jours de vie, et des enfants de mères infectées n'ayant jamais séjourné en pays endémique eux-mêmes.
Méthodes: Pour la première fois, cette revue synthétise systématiquement la littérature sur la transmission verticale de la THA. Afin de couvrir plus largement le sujet, les articles traitant de l'épidémiologie de la THA chez l'enfant et chez la femme enceinte ont aussi été inclus. Les manuels et rapports techniques de l'Organisation Mondiale de la Santé, de Médecins Sans Frontières, de l'Institut de Recherche pour le Développement, et d'un pays endémique ont été revus.
Résultats: Les publications décrivant la THA congénitale sont rares et consistent seulement en des rapports de cas uniques et de petites séries de cas. La THA congénitale est généralement considérée comme étant un événement rare, mais n'a jamais été investiguée de façon systématique. Deux publications émettent l'hypothèse que la THA congénitale serait plus fréquente que ce que l'on croit. La transmission verticale n'est pas mentionnée dans tous les manuels et publications sur la THA.
Conclusions: Le vrai risque de transmission verticale est inconnu. La prise de conscience sur la THA congénitale est insuffisante, ce qui peut conduire à un défaut de diagnostic précoce chez les nouveau-nés. Tous les manuels et protocoles opératoires devraient insister sur le dépistage systématique des femmes enceintes en zone endémique et sur l'évaluation précoce des nouveau-nés de mères infectées. Des études sur l'impact de la THA sur la fertilité et la grossesse manquent depuis longtemps.
